# Characteristic tetrapod musculoskeletal limb phenotype emerged more than 400 MYA in basal lobe-finned fishes

**DOI:** 10.1038/srep37592

**Published:** 2016-11-25

**Authors:** Rui Diogo, Peter Johnston, Julia L. Molnar, Borja Esteve-Altava

**Affiliations:** 1 Department of Anatomy, Howard University College of Medicine, USA; 2 Department of Anatomy and Medical Imaging, University of Auckland, New Zealand; 3 Structure & Motion Lab, Department of Comparative Biomedical Sciences, Royal Veterinary College, UK

## Abstract

Previous accounts of the origin of tetrapod limbs have postulated a relatively sudden change, after the split between extant lobe-finned fish and tetrapods, from a very simple fin phenotype with only two muscles to the highly complex tetrapod condition. The evolutionary changes that led to the muscular anatomy of tetrapod limbs have therefore remained relatively unexplored. We performed dissections, histological sections, and MRI scans of the closest living relatives of tetrapods: coelacanths and lungfish. Combined with previous comparative, developmental and paleontological information, our findings suggest that the characteristic tetrapod musculoskeletal limb phenotype was already present in the Silurian last common ancestor of extant sarcopterygians, with the exception of the autopod (hand/foot) structures, which have no clear correspondence with fish structures. Remarkably, the two major steps in this long process – leading to the ancestral fin anatomy of extant sarcopterygians and limb anatomy of extant tetrapods, respectively – occurred at the same nodes as the two major similarity bottlenecks that led to the striking derived myological similarity between the pectoral and pelvic appendages within each taxon. Our identification of probable homologies between appendicular muscles of sarcopterygian fish and tetrapods will allow more detailed reconstructions of muscle anatomy in early tetrapods and their relatives.

Most studies on the origin of limbs focus on fossil skeletal structures^1^,^2^,^3^,^4^,^5^, mainly because fossils usually do not preserve soft tissues, and because it is difficult to compare fish fins and tetrapod limbs as they are morphologically very different (e.g., in orientation of axes and number/configuration of muscles). Classic comparative anatomy works provided in-depth descriptions of the major rotation of the paired appendages that occurred during the early stages of the fins-limbs transition: the preaxial (radial/tibial) side, directed antero-dorsally in extant fishes such as *Polypterus*, *Latimeria* and living dipnoans, became directed antero-ventrally^6^,^7^,^8^,^9^,^10^,^11^. However, these descriptions are not always taken into account in recent works, leading to errors and terminological problems (see below, and SI). Although numerous appendicular muscles have been described in the coelacanth *Latimeria*^12^,^13^, these descriptions are often excluded from recent discussions about the fins-limbs transition because dipnoans are phylogenetically closer to tetrapods than are coelacanths^14^. Therefore, most authors consider that a transition occurred after the dipnoan-tetrapod divergence, from a very simple fin configuration with only two major muscle masses (adductor/abductor) to the highly complex tetrapod limbs that can have more than 50 muscles^15^. Accordingly, Extant Phylogenetic Bracketing^16^, one of the most powerful tools for soft tissue reconstruction, has never been used to study this fins-limbs transition^17^, despite the fact that the relationships of extant sarcoptergians have long been well established^18^.

The original data obtained from extant taxa, combined with the comparisons, that are presented in this paper will be crucial in paving the way for the use of this method in muscle reconstructions of key tetrapod and non-tetrapod sarcopterygian extinct taxa. Specifically, for this work, we obtained new musculoskeletal data from dissections, MRI scans, 3D reconstructions, and histological sections of coelacanths (*Latimeria*) and dipnoans (*Neoceratodus*) (see SI, which includes Tables S1–S4 showing all muscle-bone attachments) and combined them with data gathered during a 20-year study of the evolution, homologies and development of the muscles of vertebrates and our new observations of *Polypterus* (dissections and microCT scans: see text below, SI and Tables S5–S6). Regarding the muscular anatomy of lobe-finned fishes, the major novel contributions of this work are 1) description of new muscles; 2) re-appraisal of evolutionary origin (e.g., from ventral/abductor *vs.* dorsal/adductor masses) and identity of previously described muscles; and 3) first comprehensive comparisons of pelvic and pectoral appendages (PELA, PECA) among these and other fish and in tetrapods, leading to proposal of new names, evolutionary origins and one-to-one homology hypotheses for all muscles of these taxa (see SI for more details, in particular between the differences between our work and previous studies). We discuss our results in the context of the anatomical, developmental, and paleontological literature, including recent papers on the soft tissues of adult dipnoans^19^,^20^ and coelacanths^14^, of phylogenetically basal extant bony fishes such as *Polypterus*^21^, and works on appendicular muscle development in most gnathostome clades^11^,^12^,^22^,^23^,^24^,^25^,^26^,^27^,^28^,^29^,^30^,^31^,^32^,^33^,^34^.

## Results and Discussion

**Figure 1:**
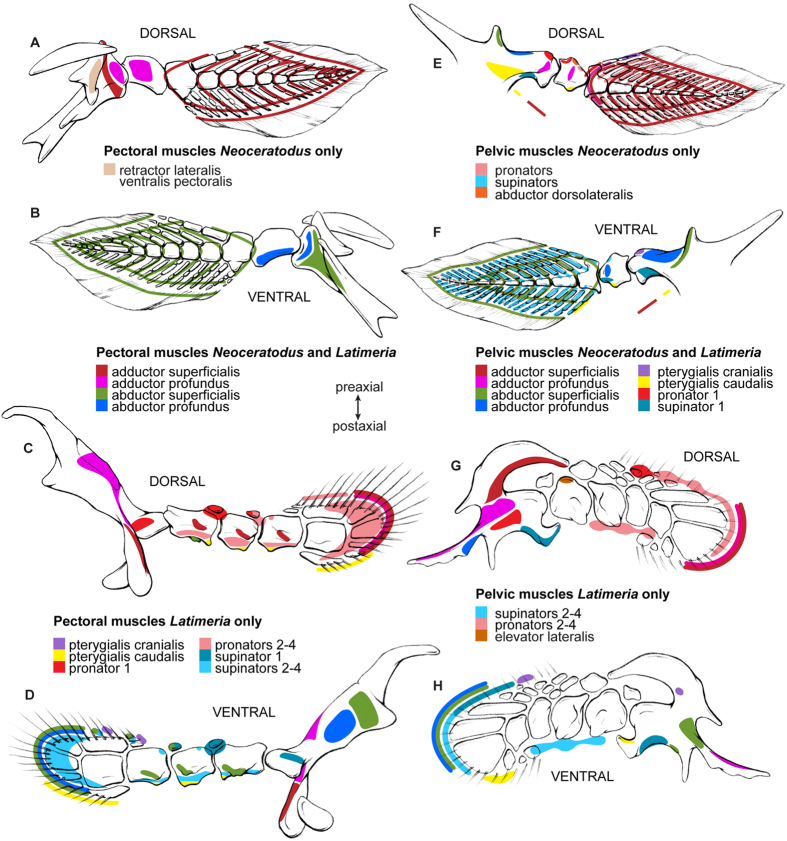
Muscle maps. Right pectoral (**A–D**) and pelvic (**E–H**) appendages of *Neoceratodus* (**A,B,E,F**) and *Latimeria* (**C,D,G,H**) in dorsal (**A,C,E,G**) and ventral (**B,D,F,H**) views. Note that the use of similar colors in the pectoral and pelvic muscles does not indicate ancestral serial homology between the structures of these paired appendages, but instead the result of derived similarity (see text).

**Figure 2:**
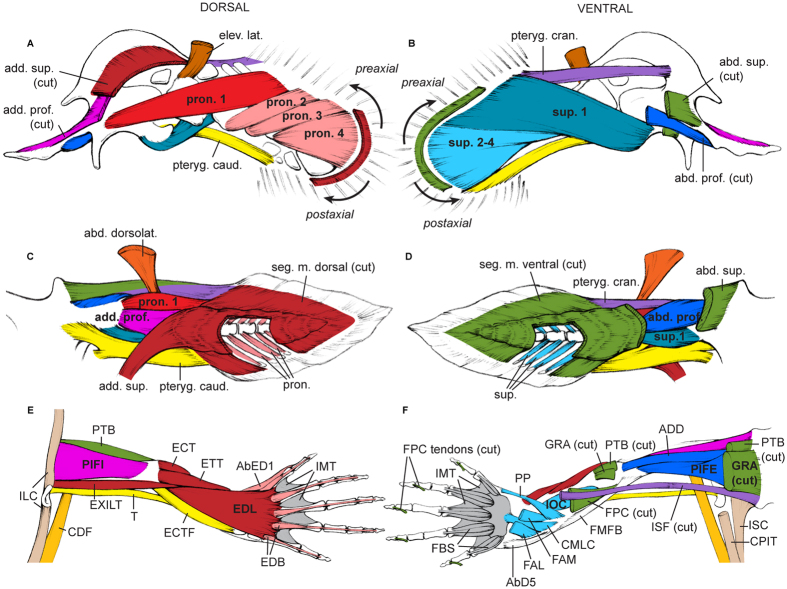
Hypotheses of hindlimb muscle homology; similar colors indicate homologous muscles. *Latimeria* (**A,B**)*, Neoceratodus* (**C,D**), and *Ambystoma* (**E,F**). Dorsal views (**A,C,E**) and ventral views (**B,D,F**). Abbreviations: abductor dorsolateralis (abd. dorsolat.), abductor profundus (abd. prof.), abductor superficialis (abd. sup.), abductor digiti minimi (AbD5), abductor et extensor digiti I (AbED1), adductor femoris (ADD), adductor profundus (add. prof.), adductor superficialis (add. sup.), contrahentium caput longum (CCL), caudofemoralis (CDF), caudalipuboischiotibialis (CPIT), extensor cruris tibialis (ECT), extensor cruris et tarsi fibularis (ECTF), extensores digitorum breves (EDB), extensor digitorum longus (EDL), elevator lateralis (elev. lat.), extensor tarsi tibialis (ETT), extensor iliotibialis (EXILT), flexor accessorius lateralis (FAL), flexor accessorius medialis (FAM), flexores breves superficiales (FBS), flexor digitorum communis (FDC), femorofibularis (FMFB), gracilis (GRA), iliocaudalis (ILC), intermetatarsales (IMT), interosseus cruris (IOC), ischiocaudalis (ISC), puboischiofemoralis externus (PIFE), puboischiofemoralis internus (PIFI), puboischiotibialis (PIT), pubotibialis (PTB), pronator profundus (PP), pubotibialis (PTB), pterygialis caudalis (pteryg. caud.), pterygialis cranialis (pteryg. cran.), segmented muscle (seg. m.), tenuissimus (T).

**Figure 3:**
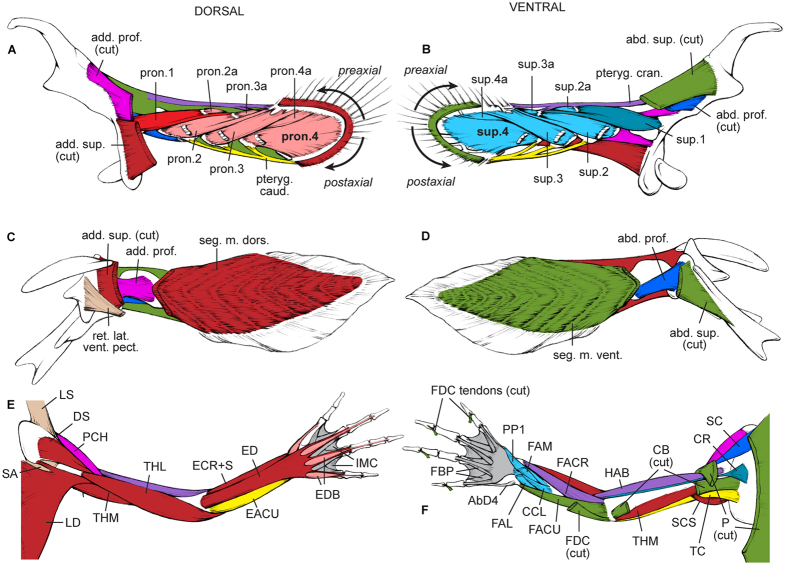
Hypotheses of forelimb muscle homology; similar colors indicate homologous muscles. *Latimeria* (**A,B**)*, Neoceratodus* (**C,D**), and *Ambystoma* (**E,F**). Dorsal views (**A,C,E**) and ventral views (**B,D,F**). Colors indicate homologous muscles. Abbreviations: abductor digiti minimi (AbD4), abductor et extensor digit 1 (AbED1), coracobrachialis (CB), contrahentium caput longum (CCL), contrahentes digitorum (CD), deltoideus scapularis (DS), extensor antebrachii et carpi ulnaris (EACU), extensor carpi radialis + supinator (ECR + S), extensor digitorum (ED), extensores digitorum breves (EDB), flexor antebrachii et carpi radialis (FACR), flexor antebrachii et carpi ulnaris (FACU), flexor accessorius lateralis (FAL), flexor accessorius medialis (FAM), flexores breves profundi (FBP), flexor digitorum communis (FDC), humeroantebrachialis (HAB), intermetacarpales (IMC), latissimus dorsi (LD), levator scapulae (LS), pectoralis (P), procoracohumeralis (PCH), palmaris profundus 1 (PP1), serratus anterior (SA), supracoracoideus (SC), triceps coracoideus (TC), triceps humeralis lateralis (THL), triceps humeralis medialis (THM), triceps scapularis medialis (TSM), coracoradialis (CR), subcoracoscapularis (SCS), retractor lateralis ventralis pectoralis (ret. lat. vent. pect.), adductor superficialis (add. sup.), retractor lateralis ventralis pectoralis (ret. lat. vent. pect.), adductor profundus (add. prof.), segmented muscle (seg. m.), abductor superficialis (abd. sup.), abductor profundus (abd. prof.), pterygialis cranialis (pteryg. cran.), pterygialis caudalis (pteryg. caud.).

Our analysis reveals that the PELA and PECA of *Latimeria* and *Ambystoma* and the PELA of *Neoceratodus* share a very similar, complex configuration of homologous (between PECAs) and topologically corresponding (between PECAs and PELAs) muscles (Tables S5–S7; Figs 1, 2 and 3). Among several striking similarities, the two limbs (PELA and PECA of *Ambystoma*) and three fins (PECA and PELA of *Latimeria* and PELA of *Neoceratodus*) share dorsal and ventral superficial muscle masses that extend from the girdles to the distal regions of the fins/limbs, a series of similar dorsal and ventral deep muscles (supinators and pronators and their derivatives), and pre- and postaxial muscles that often span more than one joint. Based on this evidence and on the strong evidence that the very simplified PECA muscle anatomy of *Neoceratodus* is a derived characteristic of dipnoans (see SI for details; see also in ref. 35), we propose that the characteristic muscle configuration of the tetrapod limbs arose through a series of stepwise changes from the last common ancestor (LCA) of extant osteichthyans to the LCA of tetrapods. The LCA of extant gnathostomes most likely had five muscles in each paired fin: ventrally, the abductor superficialis, abductor profundus and a preaxial muscle pterygialis cranialis; dorsally, the adductor superficialis and adductor profundus^36^ (Tables S5–S6). The LCA of extant bony fishes probably had the same five muscles plus a postaxial muscle (pterygialis caudalis) in both the PELA and PECA, because the plesiomorphic extant osteichthyan *Polypterus* (see in ref. 21 and our observations) and *Latimeria* share the presence of this muscle in each appendage (Tables S5–6, Figs 1 and 3). This postaxial muscle (“zonopropterygialis” in *Polypterus* PECA sensu Wilhelm *et al.*^21^; present in both its PECA and PELA according to our observations) and the preaxial muscle pterygialis cranialis (“coracometapterygialis I and II” in *Polypterus* PECA sensu Wilhelm *et al.*^21^; present in both its PECA and PELA according to our observations) are thought to be derived from the dorsal (adductor/‘levator’) and ventral (abductor/‘depressor’) fin musculature, respectively^36^. However, in some fishes ventral muscles may be differentiated postaxially, and dorsal muscles preaxially^34^.

**Figure 4:**
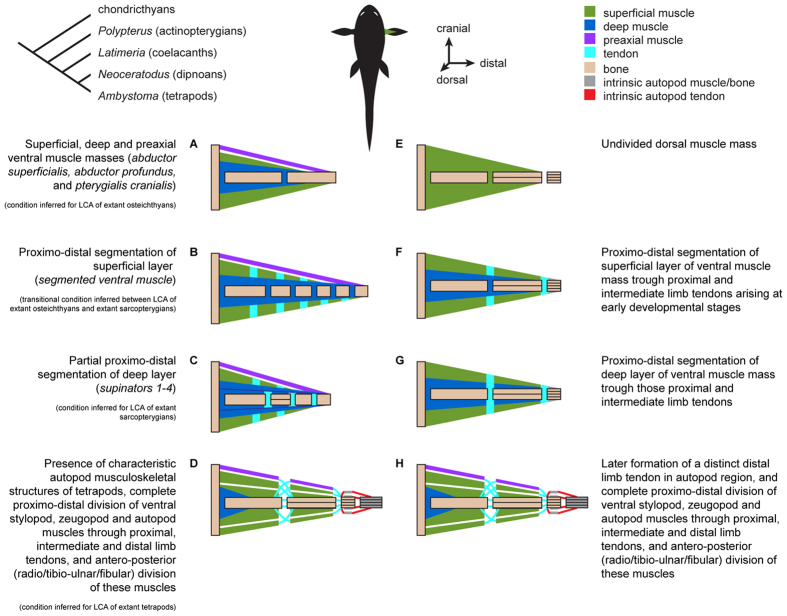
Evolutionary and developmental transitions leading to the modern adult tetrapod limb. Evolutionary transitions in adult morphology exemplified by ventral musculature, based on the present paper (**A**): LCA of extant osteichthyans; (**B**): stem sarcopterygians; (**C**): LCA of extant sarcopterygians; (**D**): LCA of extant tetrapods: see cladogram on top left), and developmental transitions from early stages to adult morphology in tetrapods (**E–H**), exemplified by ontogeny of ventral musculature in chicken hindlimb (based on Kardon^26^). All images show dorsal views with dorsal muscles (therefore including the dorsal, postaxial muscle pterygialis caudalis) removed.

A striking implication of our synthesis is that the LCA of extant sarcopterygians probably already had the basic tetrapod limb phenotype in both PECA and PELA, with the exception of the characteristic tetrapod autopod (hand/foot) (Tables S5–S7; Fig. 4C). Specifically, this LCA probably had at least two layers of adductor and abductor muscles that were partially segmented proximo-distally at the level of each joint. That is, the dramatic changes between the LCA of extant bony fishes and the LCA of extant sarcopterygians affected in a markedly similar way the ventral and dorsal sides of both the PECA and PELA. In particular, the deep musculature (adductor profundus dorsally; abductor profundus ventrally) gave rise to a series of smaller muscles (pronators dorsally; supinators ventrally) (Figs 1, 2, 3 and 4; Tables S5–S7). An illustrative example of the pronounced overall PECA-PELA similarity of sarcopterygians is the almost identical configuration of the *Latimeria* PELA and PECA, which is in turn strikingly similar to that of the *Neoceratodus* PELA (Tables S5–S6; Figs 2 and 3). Because of this marked PECA-PELA similarity, most hypotheses of homology shown in Tables S5–S6 are straightforward. An extensive account of the rationale and evidence behind each of the hypotheses shown in these tables is provided in the SI.

As shown in Fig. 4, the inferred order of phylogenetic events leading to the origin of tetrapod limbs is very similar to that of the ontogeny of the limbs of extant tetrapods. Moreover, the rotation of the paired appendages (internal rotation sensu human anatomy) that occurred over the fins-limbs transition, turning the ventrolateral abductor (‘depressor’) fin musculature towards the body to become the limb ‘flexor musculature’ in tetrapods, is also paralleled by a similar rotation during the ontogeny of tetrapods such as salamanders^21^. The chief exception to this developmental-phylogenetic similarity is that the preaxial and postaxial muscles pterygialis cranialis and caudalis were differentiated evolutionarily long before the appearance of clear tendinous intersections segmenting proximo-distally the main abductor/adductor fin musculature. In contrast, such intersections appear at very early stages of tetrapod limb development, before any observable antero-posterior (i.e., radio-ulnar or tibio-fibular) division of the musculature is evident (although recent studies of myobast migration in mice suggest that such an antero-posterior division of the limb musculature might actually happen earlier in development than previously thought: Sevan Hopyan, pers. comm.). However, this difference makes sense from a biomechanical perspective: segmented or divided muscles that cross only one joint are only effective when the fin skeleton is elongated and segmented proximo-distally into numerous bones connected by numerous and/or more mobile joints, as is the case in lobe-finned fishes but not in most other fishes (Fig. 4B). Accordingly, early morphogenesis of limb skeletal cartilages and joints in tetrapods is associated with early morphogenesis of proximal and intermediate tendons lying in the region of the major limb joints: the elbow/knee and wrist/ankle joints, respectively (Fig. 4F). In fact, this is probably a chief developmental constraint in extant tetrapods, as such limb tendons likely can only develop ontogenetically in the neighborhood of joints^26^. One interesting point is that, contrary to what is usually seen in the ontogeny of extant tetrapods, in sarcopterygian fishes such as *Latimeria* the intersections of the superficial layer mainly lie *at the level* of the major fin bones, and not *between* these bones, i.e. in the region of the joints connecting them (Figs 1 and 4B,C).

Also interestingly, some aspects of our evolutionary hypothesis (Fig. 4A–D) are similar to those proposed more than 120 years ago by Gadow^37^. He suggested that muscles running all the way from the axial skeleton/musculature and/or girdles to the distal region of the fins became proximo-distally partitioned in the region of major joints - particularly those related to the overall internal rotation of the fins - during the fins-limbs transition. This view, which is supported by the present work, contradicts the statements of more recent works, particularly paleontological ones. For example, in Bishop’s detailed reconstruction of the shoulder/arm/forearm muscles of a stem tetrapod it was assumed that ancestrally these muscles did not cross more than one joint^17^. However, it should be noted that a few paleontologists did propose a proximo-distal partition of muscles that originally crossed more than one joint, during the fins-limbs transition, as suggested by Gadow (see, e.g. in ref. 35).

Most authors agree that the tetrapod stylopod and zeugopod bones are homologous with the proximal bones of sarcopterygian fins, but whether the tetrapod autopodia are neomorphic structures or include structures homologous to specific fin structures remains controversial^2^,^38^,^39^,^40^,^41^,^42^,^43^,^44^. Some evidence from soft tissue development favours the neomorphic hypothesis. For example, during tetrapod development the distal tendon primordium that gives rise to most tendons of the intrinsic hand/foot muscles appears later than the primordia of the proximal and intermediate tendons associated with girdle, stylopod (arm/thigh) and zeugopod muscles (Fig. 4H). Additionally, there are significant differences between the morphogenesis of the proximal/intermediate tendons *vs.* the distal tendon^26^ (see also, e.g., more recent works from Schweitzer’s group, reviewed in Huang *et al.*^27^). While the segregation of the primordia of the former tendons depends on interactions with muscle, the distal tendons 1) develop by a two-step process in which their primordium segregates into various tendon blastemas – each associated with a digit – that in turn subdivide into individual tendons; 2) develop mainly in spatial isolation from, and likely independently of interactions with, the muscles to which they will attach; and 3) express the transcription factors *six-1* and *six-2* and the *eph*-related receptor tyrosine kinase *cek-8*, while proximal/intermediate tendons do not (reviewed by Kardon^26^). These developmental data, combined with our comparative anatomical data, support the idea that the overall musculotendinous configuration of the hand/foot constitutes a tetrapod evolutionary novelty^26^, probably acquired later in evolution than were most of the girdle/stylopod/zeugopod muscles (Fig. 4D).

A recent compilation of comparative anatomical, paleontological and developmental data strongly suggests that the PECA and PELA were markedly different from each other anatomically in the earliest fishes that had both, and that their most proximal regions (i.e., pelvic *vs.* pectoral girdles) have remained anatomically, developmentally and genetically quite different^45^,^46^,^47^. In contrast, the co-option of various similar genes in the development of the more distal, and phylogenetically more recent, stylopod/zeugopod and particularly autopod regions of the PECA and PELA of tetrapods led to a marked *derived* anatomical and developmental similarity between these structures in both appendages (i.e., a ‘similarity bottleneck’ sensu Diogo *et al.*^46^, Diogo and Molnar^48^, and sensu the present work). These more distal limb regions, principally the autopodia, display developmental patterns that are quite different from those of the fins of plesiomorphic gnathostomes^41^, and of more proximal limb regions in tetrapods. This information agrees with the notes in the previous paragraph regarding the distal *vs.* proximal/intermediate tendons (Fig. 4B) and with data on the development and genetic networks of tetrapod limbs^47^. However, it remained an open question whether such a co-option and/or other (e.g., functional/topological) factors leading to the PECA-PELA similarity bottlenecks might have occurred even before the rise of tetrapods. Our results suggest that there was in fact a second, much earlier major similarity bottleneck between the muscles of the PECA and PELA: during the transition from the LCA of extant bony fishes to the LCA of extant sarcopterygians. This latter LCA probably already displayed striking muscular similarities not only between the dorsal and ventral sides of each fin, but also between the two PECA and two PELA, thus essentially having eight copies of the same highly complex configuration. This condition is exemplified by *Latimeria,* in which 14 PECA muscles have clear, straightforward one-to-one topological correspondences with PELA muscles, and the dorsal muscles of each fin have clear one-to-one correspondences with ventral muscles on the same fin (Figs 2 and 3; Tables S5–S7). In contrast, the similarity between six of the muscles of each paired appendage of plesiomorphic actinopterygians and osteichthyans, such as *Polypterus,* is mainly due to the fact that these fins display a very simple, basic condition that was acquired much earlier in gnathostome evolution: the presence of poorly differentiated deep and superficial abductor/adductor masses^36^ (Fig. 4; Tables S5–S6).

Our study therefore allows us, for the first time, to propose a detailed scheme of topological correspondences between all PECA *vs.* PELA muscles, including girdle/stylopod muscles, based on the same empirical comparative, evolutionary, and developmental data used for the homology hypotheses (Table S7). Such schemes have previously been attempted, mostly in the 19^th^/early 20^th^ centuries, but they were strongly biased by the old Romantic ‘archetypal’, idealistic view of evolution^46^. As seen in Table S7, the topological correspondences inferred here between the girdle/stylopod muscles of the PECA and PELA are, in both salamanders and humans, mainly between groups of muscles, without clear one-to-one equivalences, while those between the zeugopod/autopod muscles are mainly one-to-one. Therefore, our results reinforce the idea that muscles associated with the pectoral and pelvic girdles have remained more different from each other since the appearance of these appendages in basal gnathostome fishes in comparison to the more distal muscles, which were affected by similarity bottlenecks during the transitions leading to sarcopterygians and then to tetrapods.

In summary, the fins-limbs transition was a long, stepwise process, and the characteristic tetrapod musculoskeletal limb configuration was very likely present in the Silurian LCA of extant sarcopterygians, more than 400 MYA. In addition to the fact that proximal bones and numerous muscles of the paired appendages of *Latimeria* and *Neoceratodus* have clear homologues in tetrapods, the absolute numbers of muscles in each appendage suggest that the muscle configuration of extant sarcopterygian fishes is, in fact, more similar to that of tetrapods than to that of any other extant fishes. Chondrichthyans such as sharks have five muscles in each paired appendage (Total = 10) and plesiomorphic osteichthyans such as *Polypterus* have six pectoral and six pelvic (T = 12), while *Latimeria* has 20 and 15 (T = 35), *Neoceratodus* five and 25 (T = 30) and anatomically plesiomorphic tetrapods such as *Ambystoma* have 48 and 59 (T = 107), respectively (Tables S5–S6; see cladogram of Fig. 4). If we exclude intrinsic hand/foot muscles, which do not seem to be directly homologous to any specific fish muscles, *Ambystoma* has 28 and 27 (T = 55), only 20 more than the number found in *Latimeria*, so the difference between *Polypterus* and *Latimeria* (35–12 = 23) is, strikingly, larger than that between *Latimeria* and *Ambystoma*. Moreover, the data provided here point out that the major transitions that led to the characteristic phenotype of tetrapod limbs (one leading to sarcopterygians and the other to tetrapods) corresponded to the two major similarity bottlenecks that led to the striking derived myological similarity between the PECA and PELA. Finally, by providing one-to-one homology hypotheses for each muscle of the paired appendages of all these taxa, this work lays the foundation for the use of Extant Phylogenetic Bracketing in musculoskeletal reconstructions in paleontological studies on the origin/early evolution of limbs.

## Materials and Methods

### Anatomical studies

Two formalin fixed adult specimens of *Neoceratodus forsteri* (JVM-I-1051NC, JVM-I-1052NC) were donated by Macquarie University, Australia, and dissected under magnification. A formalin fixed adult specimen of *Latimeria chalumnae* (SZ 10378, or CCC161 according to Nulens *et al.*^49^) was dissected at the Institüt für Evolution und Ökologie, Universität Tübingen, and serial histological sections of a *Latimeria* embryo (CCC162.11; see Nulens *et al.*^49^) including pectoral fin and girdle were also examined in Tübingen. MRI scans of *Neoceratodus forsteri* and *Latimeria chalumnae* were provided by the Digital Fish Library, UCSD (www.digitalfishlibrary.org). Three frozen adult specimens of *Polypterus senegalus* (HUPS1, HUPS2, HUPS3) from the collection of Rui Diogo’s lab, Howard University, USA, were dissected under magnification. One formalin fixed adult specimen of *Polypterus delhezi* (JVM-I-49PD) was donated by the University of Auckland, New Zealand, to perform micro-CT scans (see Tables S5 and S6). Images were resized in ImageJ (NIH) and 3D reconstruction of fin skeleton and muscles was performed with Amira 5.2.1 (Visage Imaging) with manual segmentation of structures. Information about *Ambystoma* is from our previous works on the development, regeneration, and adult anatomy of the limb muscles of salamanders^23^,^50^,^51^,^52^. No experiments on live vertebrates were performed for this study.

### Formulation of homology hypotheses

In Tables S5–S6 and Figs 1, 2 and 3 we present one-to-one homology hypotheses for the muscles of the paired appendages across five major extant gnathostome clades: chondrichthyans (shark *Squalus*), as the extant sister-group of osteichthyans (bony fishes); actinopterygians (bichir *Polypterus*), as the extant sister-group of sarcopterygians; coelacanths (*Latimeria*), as the extant sister group of dipnoans plus tetrapods; dipnoans (*Neoceratodus*); and tetrapods (*Ambystoma*), as salamanders are, anatomically, the most plesiomorphic extant tetrapods (i.e., the most similar to the LCA of extant tetrapods^12^,^33^,^36^). As explained in the main text, our hypotheses of homology between fin muscles in *Latimeria* and *Neoceratodus* (Tables S5–S6) are very straightforward because three out of the four fins studied have very similar muscle configurations. An entire book could be written describing each and every point supporting the homologies of each muscle of each paired appendage of each of the three taxa listed on those tables. Therefore, here we summarize the major points supporting key homology hypotheses. The same points can be applied to support similar homology hypotheses between other muscles. These hypotheses combine developmental, anatomical, and paleontological evidence and multiple cross-comparisons with other muscles from the same and from other paired appendages in different taxa, and embryonic primordia, following strict standards of homology such as: 1) positional equivalence, determined by bony attachments; 2) special quality, determined by, e.g., the orientation of fibers and innervation of muscles; 3) transition, determined by paleontological and/or developmental evidence of intermediate conditions; and 4) congruence, determined by applying the previous criteria to adjacent muscles and muscles of both the dorsal and ventral sides of each appendage and of the two paired appendages (i.e., pectoral *vs.* pelvic). For example, regarding the use of paleontological data, the homology hypotheses shown in Table S5 and Fig. 2 are consistent with microanatomical evidence that the humerus of the early tetrapodomorph fish *Eusthenopteron* had osteological correlates of a muscle corresponding to pronator 1 in *Latimeria*^53^. Regarding the use of ontogenetic data, an illustrative example concerns the pterygialis cranialis of the pelvic fin of *Latimeria* and *Neoceratodus*, which is similar to the pelvic muscle ischioflexorius of salamanders because developmental evidence supports the idea that both the pterygialis cranialis of fish and the ischioflexorius of tetrapods are derived from the ventral embryonic muscle mass^23^,^36^. In fact, distally the ischioflexorius includes the ancestral leg muscle flexor cruris et tarsi tibialis, which is a preaxial muscle (like the pterygialis cranialis) that corresponds topologically to the preaxial forearm flexor antebrachii et carpi radialis muscle of the salamander forelimb^34^ (Figs 2 and 3; Table S5–S6). Moreover, in tetrapods such as salamanders the flexor antebrachii et carpi radialis differentiates from a different primordium than do the more ulnar/postaxial muscles flexor antebrachii et carpi ulnaris and flexor digitorum communis, suggesting that the former preaxial forearm muscle, as well as the corresponding preaxial leg muscle flexor cruris et tarsi tibialis, derive from the pterygialis cranialis muscles of the pectoral and pelvic appendages, respectively. Accordingly, the superficial and postaxial muscles of the ventral zeugopod, such as the forearm muscle flexor antebrachii et carpi ulnaris and the leg muscle flexor cruris et tarsi fibularis, derive from the fish abductor superficialis, as do the flexor digitorum communis and other ventral superficial muscles.

Similar reasoning leads to the hypothesis of homology between the pelvic fin muscle pterygialis caudalis and the tetrapod muscles tenuissimus and extensor cruris et tarsi fibularis. The latter muscle is the mirror image (dorsal instead of ventral, and fibular instead of tibial) of the flexor cruris et tarsi tibialis in other salamanders which, because of its evolutionary and developmental history, is probably included in the tenuissimus of *Ambystoma*^33^. Therefore, because both muscles are probably derived from a single ancestral muscle, lie on the postaxial side of the limb, and develop from the dorsal muscle mass, they are probably derived from the pelvic postaxial muscle pterygialis caudalis. The same argument supports homology between the pectoral fin muscle pterygialis caudalis and part of triceps plus the extensor antebrachii et carpi ulnaris of tetrapods. The latter muscle is the mirror image of the flexor antebrachii et carpi radialis^21^, corresponding topologically to the extensor cruris et tarsi fibularis of the tetrapod hindlimb^48^.

The homology between the retractor lateralis ventralis pectoralis of fish and the serratus anterior and levator scapulae of salamanders rests on the fact that these are the only muscles in the two taxa that connect the axial skeleton to the pectoral girdle, i.e., they are primaxial muscles (Fig. 2; Table S5). Homology between the retractor lateralis ventralis pectoralis of fishes and the serratus anterior complex of tetrapods has been proposed by previous authors^54^,^55^. The caudofemoralis in *Ambystoma*, levator lateralis in *Latimeria,* and abductor dorsolateralis in *Neoceratodus* are included in the abaxial/primaxial group of muscles because all originate from the axial skeleton and/or axial muscles (Table S5). However, we do not conclude that these muscles are directly homologous because the levator lateralis in *Latimeria* seems to be part of the dorsal musculature while the tetrapod caudofemoralis is part of the ventral musculature^23^,^48^.

The present work is the first to propose that the abductor and adductor superficialis are homologous with the superficial muscles that extend all the way from the body wall or girdles to the autopodia in tetrapods. However, the same idea was presented in a more theoretical way by Gadow^37^. The author suggested that muscles running all the way from the axial skeleton/musculature and/or the girdles to the distal region of the fins became proximo-distally partitioned in the region of major joints during the fins-limbs transitions. The homology hypotheses in the present work combine Gadow’s evolutionary scenario with developmental and comparative data that were not available in his time^37^. For instance, developmental data for *Ambystoma* show that the superficial layer of the ventral muscles of the pectoral girdle, arm and forearm comprise the pectoralis, flexor digitorum communis, flexor antebrachii et carpi ulnaris, coracobrachialis, humeroantebrachialis and flexor antebrachii et carpi radialis^23^. Therefore, we propose that the fish abductor superficialis gave rise to and is homologous with all of these developmentally ventral superficial muscles. The only exceptions are the humeroantebrachialis and flexor antebrachii et carpi radialis in *Ambystoma* which, as explained above, most likely correspond to the pterygialis cranialis, derived from the superficial ventral (abductor) musculature, in fish (Table S5; Fig. 2).

Also on the basis of topology and developmental history (in salamanders), we propose that the second most superficial ventral muscle of the pectoral fin (abductor profundus) is homologous with the second most superficial ventral pectoral muscle of salamanders, developmentally (supracoracoideus: Table S5; Figs 2 and 3). In fact, in its attachments, fibre orientation and overall configuration the abductor profundus of the dipnoan pectoral fin is strikingly similar to the supracoracoideus of salamanders (Fig. 2). Both are short, parallel-fibred triangular muscles running from the ventral aspect of the pectoral girdle near the shoulder joint to the ventral surface of the proximal humerus (Fig. 2). Likewise, the supinator 1, which is the most proximal of the deeper muscles of the pectoral fin in *Latimeria* and connects the girdle to both the first and second fin elements, is probably homologous with the coracoradialis, which is the most proximal of the developmentally deeper muscles of salamander and originates from the girdle and runs along the humerus to insert onto the radius (Table S5; Figs 2 and 3). The same reasoning supports homology between the remaining pronators of the pectoral fin in *Latimeria* and the more distal deep ventral muscles of the salamander (Table S5). The same topological reasoning was applied to reach the homology hypotheses proposed for the adductor superficialis, adductor profundus and pronators of the pectoral fin, and also for the abductor and adductor superficialis, abductor and adductor profundus, supinators and pronators of the pelvic fin because, as explained in the main text, the four paired appendages of each taxon essentially include eight copies of the same model. Among the relatively straightforward homology hypotheses between the fish and tetrapod muscles, the most speculative concern the autopodial muscles. As shown in Tables S5 and S6, none of the intrinsic autopodial muscles of tetrapods seems to be present as a separate, distinct structure in extant sarcopterygian fish. However, future work on intermediate fossils could reveal that some supinators and/or pronators in fish directly correspond to tetrapod autopodial muscles.

## Additional Information

**How to cite this article**: Diogo, R. *et al.* Characteristic tetrapod musculoskeletal limb phenotype emerged more than 400 MYA in basal lobe-finned fishes. *Sci. Rep.* **6**, 37592; doi: 10.1038/srep37592 (2016).

**Publisher’s note:** Springer Nature remains neutral with regard to jurisdictional claims in published maps and institutional affiliations.

## Supplementary Material

Supplementary Information
