# Supplementary material for: Characteristic tetrapod musculoskeletal limb phenotype emerged more than 400 MYA in basal lobe-finned fishes
Source: Sci Rep. 2016 Nov 25;6:37592. doi: 10.1038/srep37592 (PMC5122878; doi:10.1038/srep37592)
Supplement: Supplementary Information [file srep37592-s1.docx]

Supplementary Materials for

**Characteristic tetrapod musculoskeletal limb phenotype emerged more than 400 MYA in basal lobe-finned fishes**

R Diogo*, P Johnston*, JL Molnar*, B Esteve-Altava

correspondence to: rui.diogo@howard.edu

**This PDF file includes:**

Supplementary Results and Discussion

1. Differences between our results and previous anatomical studies on *Latimeria* and *Neoceratodus*.
2. Discussion on muscle anatomy of pectoral fin of *Neoceratodus*.

Supplementary Figures S1 to S6

Supplementary Tables S1 to S7

**Supplementary Results and Discussion**

Differences between our results and previous anatomical studies on *Latimeria* and *Neoceratodus*

Very few studies have described the muscles of the paired appendages of *Latimeria* and *Neoceratodus* in detail. This study is the first to include both dissections and MRI scans of the muscles of both taxa. In addition, we compare the muscles between the two taxa and with those of the salamander *Ambystoma*. Previous studies on *Neoceratodus*^6,8^ reported only two muscle masses on the pectoral appendage: an adductor mass subdivided into superficial and deep muscles, and an abductor mass also subdivided into superficial and deep muscles. Diogo and Abdala^15^ also described a muscle “connecting the cranial rib to pectoral girdle” which corresponds to the retractor lateralis ventralis pectoralis sensu the present work (Tab S5). Because a similarly simple configuration is found in the fins of other extant dipnoans (*Protopterus* and *Lepidosiren*) which are the closest extant relatives of tetrapods, most authors agreed that this configuration was shared by the LCA of extant dipnoans and tetrapods as well^15^. The results of the present study of the pectoral appendage of *Neoceratodus* agree with those of Diogo and Abdala^15^.

Concerning the muscles of the pelvic appendage of *Neoceratodus*, the most detailed previous descriptions are those of Young *et al.*^56^ and Boisvert *et al.*^19^. The results of the current study mainly agree with those of Young *et al.*^56^. However, the authors did not include comparisons with other fishes, so the nomenclature they used was mainly descriptive. For instance, Young *et al.*^56^ describe 'radial flexors' and 'lepidotrichial flexors' as separate muscles, but these structures clearly correspond topologically to part of the segmented muscles abductor superficialis and adductor superficialis found in the pectoral fin of *Neoceratodus* and in both the pectoral and pelvic fins of *Protopterus*^20^ and *Lepidosiren*^8^ (Tab S5, S6; Figs. 2, 3; see main text). Also, we disagree with some of the muscle groups assigned by Young *et al.*^56^. For instance, their “deep ventral adductor-depressor” appears to be a ventral muscle and thus part of the abductor, and not of the adductor, musculature. It originates mainly from the ventral side of the girdle and inserts exclusively on the ventral side of the fin, as shown in Fig. 1 and in their Fig. 12.

Boisvert *et al.*^19^ do compare the pelvic appendicular muscles of *Neoceratodus* with those of other taxa (*Ambystoma* and *Latimeria*, the same taxa used for the present work). However, their discussion is mainly focused on skeletal rather than muscle homologies, and they do not discuss the broader evolutionary and phylogenetic implications of their muscle comparisons. We agree with some but not all of the authors’ muscle homology hypotheses. For instance, they suggest that the ventral pelvic muscle "superficial ventrolateral adductor" (sensu Young *et al.*^56^; lateral part of pterygialis caudalis sensu the present work) in *Neoceratodus* is homologous with the muscle “puboischiotibialis” (gracilis) in *Ambystoma*, which is actually a dorsal muscle. Similarly, they suggest that the dorsal pronators 1-3 in *Latimeria* are homologous with the ventral muscle “deep ventral adductor depressor” (supinator 1) in *Neoceratodus*.

The only previous detailed account of the pectoral and pelvic muscles of *Latimeria* is Millot & Anthony's monograph^12^. The monograph is detailed and beautifully illustrated, but it is not very accessible because it is in French, uses uncommon terminology, and is not easy to find. Like Young *et al.*^56^, the authors did not make detailed comparisons with other fishes and tetrapods or discuss the origin and early evolution of limbs. The results of the present work agree with those of Millot & Anthony^12^ with two exceptions. First, the authors described a “pronator 5” and a “supinator 5” in the pectoral fin, but these muscles appear to be very different from the other pronators and supinators. While the latter are short muscles that run diagonally between the pre- and post-axial edges and span 1-2 mesomeres, their “pronator 5” and “supinator 5” are very large, long muscles that run along the postaxial edge of the fin and seem to correspond to the postaxial muscles of fishes such as *Polypterus* (“pterygialis caudalis,” Figs. 3 and S2B). Second, we found several additional muscles in the pectoral fin: small, pre-axial muscles that span 2 mesomeres (pronators 2a, 3a and 4a and supinators 2a, 3a and 4a; Figs. 3 and S5).

Miyake *et al.*^13^ recently re-described the pectoral muscles of *Latimeria*. The authors disagreed with Millot & Anthony^12^ on several points, including the orientation of the fin axis, the number and placement of pronators and supinators, and the attachments of muscles onto the dorsal and ventral processes of the axial elements of the fin. According to Miyake et al., the “ventral ridges” of Millot & Anthony *(“crochets du bord inferior”)* are oriented more laterally (presumably in neutral position). We agree that the fin may be habitually held in this position during life, as mentioned in the main text we agree with the anatomical axes of Millot & Anthony*.* Miyake *et al.* found nine pronators and nine supinators; as stated above, we found seven of each while Millot & Anthony found five of each. Miyake *et al.* also state that, contrary to Millot & Anthony*,* the pronators are located on the lateral side and the supinators on the mesial side. However, this distinction seems to relate to the position of the fin rather than to the identity of the muscles. Both Figure 3a of Miyake *et al.* and Plate LXIX of Millot & Anthony seem to show the pronators on the same side of the fin as the abductors. Finally, Miyake *et al.* state that a portion of the first pronator and supinator muscles is attached to the ridges on the humerus, but they do not mention or figure an attachment of the superficial adductors and abductors onto these ridges. On the contrary, like Millot & Anthony*,* we found that the superficial adductors and abductors were attached to these ridges via the tendinous intersections (Fig. S5).

Discussion on muscle anatomy of pectoral fin of *Neoceratodus*

To discuss the configuration of the paired fins of the LCA of extant sarcopterygians, we must consider whether the very simplified muscle anatomy of the pectoral fin of *Neoceratodus* is most likely representative of the LCA of dipnoans + tetrapods or a derived characteristic of dipnoans. Several lines of evidence indicate that the non-differentiation of the pterygialis caudalis and pterygialis cranialis in the pectoral fin of *Neoceratodus*, as well as of other muscles inferred to have been acquired during the transitions from the LCA of bony fishes to the LCA of sarcopterygians, such as the pronators and supinators, results from secondary simplification of this fin. First, pronators and supinators are present in the pectoral fin of the phylogenetically most basal extant sarcopterygian, *Latimeria*, and they appear to be homologous with muscles/muscle groups of the deep forelimb musculature of tetrapods (Figs. 1-4; Table S5, S7). Second, the pelvic fin of *Neoceratodus* contains many more muscles than the pectoral fin, including muscles that appear to be homologous with pronators and supinators of coelacanths and tetrapods (deep, segmentally arranged muscles with a fibre direction diagonal to the main axis of the fin), and the pterygialis cranialis and pterygialis caudalis of coelacanths (strap-like muscles with parallel fibres running the length of the pre- and postaxial edges of the fin). Third, it is consensually accepted that the fins of the two other extant dipnoan species (*Protopterus*, *Lepidosiren*) were secondarily simplified.

The pectoral fin muscles of *Lepidosiren* are in fact very similar to the pectoral fin muscles of *Neoceratodus*, including a primaxial retractor, lateralis ventralis pectoralis, that corresponds to the tetrapod 'serratus' (sensu Humphry^8^), adductor superficialis ('latissimus dorsi'), abductor superficialis ('pectoralis'), abductor profundus, and adductor profundus ('coracobrachialis'). The pelvic fin of *Lepidosiren* displays an even more extreme case of secondary simplification, having only two muscles, one adductor and one abductor^8^. Such a simplified configuration is also found in the pelvic fin of *Protopterus*: the 'protractor + anterior circumradials' and the 'retractor + posterior circumradials' described by King & Hale^20^ are actually bundles of fibres of the continuous adductor/abductor muscles that are just slightly separated superficially by connective tissue attaching onto the skin. Fourth, the muscle configuration of the pelvic fins of *Protopterus* and *Lepidosiren* just described is strikingly similar to that of tetrapod limbs at early developmental stages (Fig. 4) and in some adult tetrapods with marked secondary limb reduction, which have only adductor and abductor limb muscle masses^57^. Finally, recent developmental studies of the *Neoceratodus* pectoral fin showed that at early stages there is a radius and an ulna, as is the case in basal adult sarcopterygians (e.g., *Sauripterus*) and likely in adults of the extinct dipnoan genus *Pentlandia*; at later developmental stages these two cartilages fuse into a single element^38,58^, mirroring the evolutionary trend toward secondarily simplification of the fins in dipnoans.

**REFERENCES**

56. Young, G. C., Barwick, R. E. & Campbell, K. S. W. in *Pathways in Geology: Essays in Honour of Edwin Sherbon Hills* (ed. LeMaitre, R. W.) 59–75 (Blackwell Scientific, 1989).

57. Abdala, V., Grizante, M. B., Diogo, R., Molnar, J. & Kohlsdorf, T. Musculoskeletal anatomical changes that accompany limb reduction in lizards. *J Morphol* **276,** 1290–1310 (2015).

58. Jude, E., Johanson, Z., Kearsley, A. & Friedman, M. Early evolution of the lungfish pectoral-fin endoskeleton: evidence from the Middle Devonian (Givetian) *Pentlandia macroptera*. *Front. Earth Sci.* **2,** (2014).


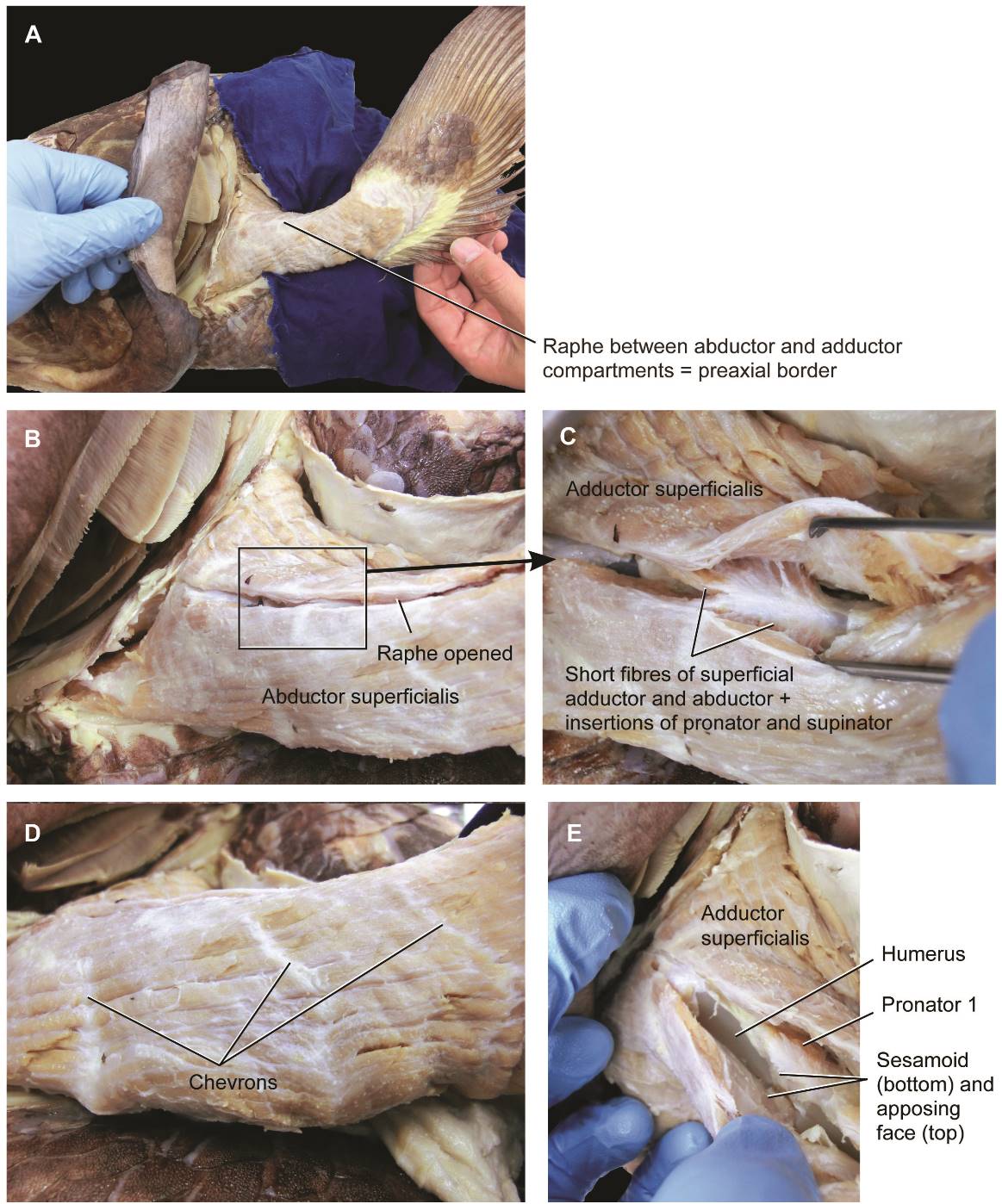


Fig. S1. Example of the type of detailed dissections done for the present work (specific details about the musculoskeletal structures of each appendage of *Latimeria* and *Neoceratodus* are given in the main text and its Tab S1-S6 and Figs. 1-3): a lateral view of the left pectoral fin of *Latimeria.* A) Intact fin with skin removed; B) raphe opened; C) superficial adductor and abductor retracted to show insertions of pronators and supinators; D) close-up of tendinous chevrons (intersections) dividing abductor superficialis; E) pronator 1 retracted to show sesamoid bone on preaxial edge of fin.


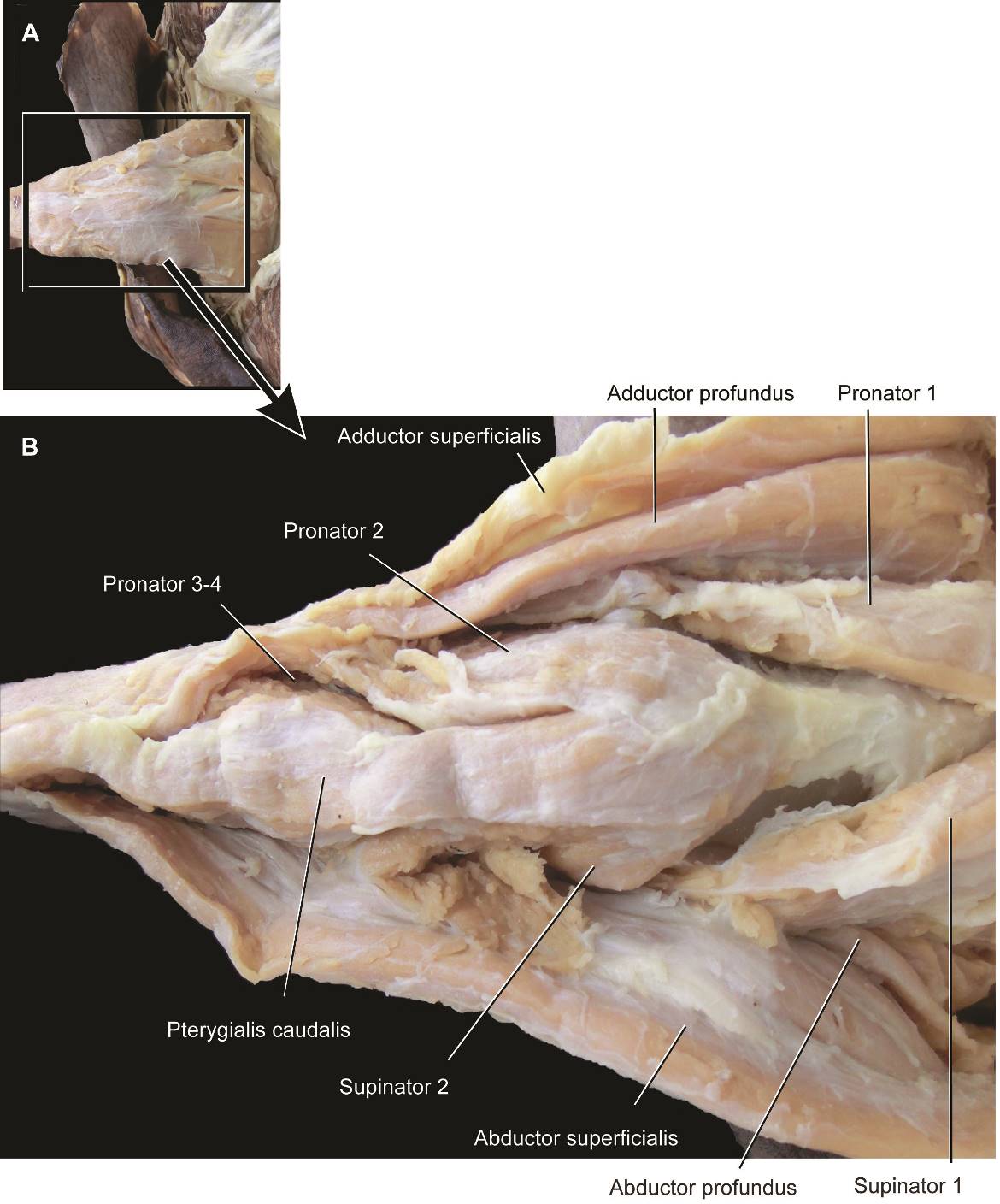


Fig. S2. Another example of the type of detailed dissections done for the present work (specific details about the musculoskeletal structures of each appendage of *Latimeria* and *Neoceratodus* are given in the main text and its Tab S1-S6 and Figs. 1-3): a medial view of the left pectoral fin of *Latimeria.* A) Intact fin with skin removed; B) Adductor and abductor superficialis retracted to show deeper muscles.


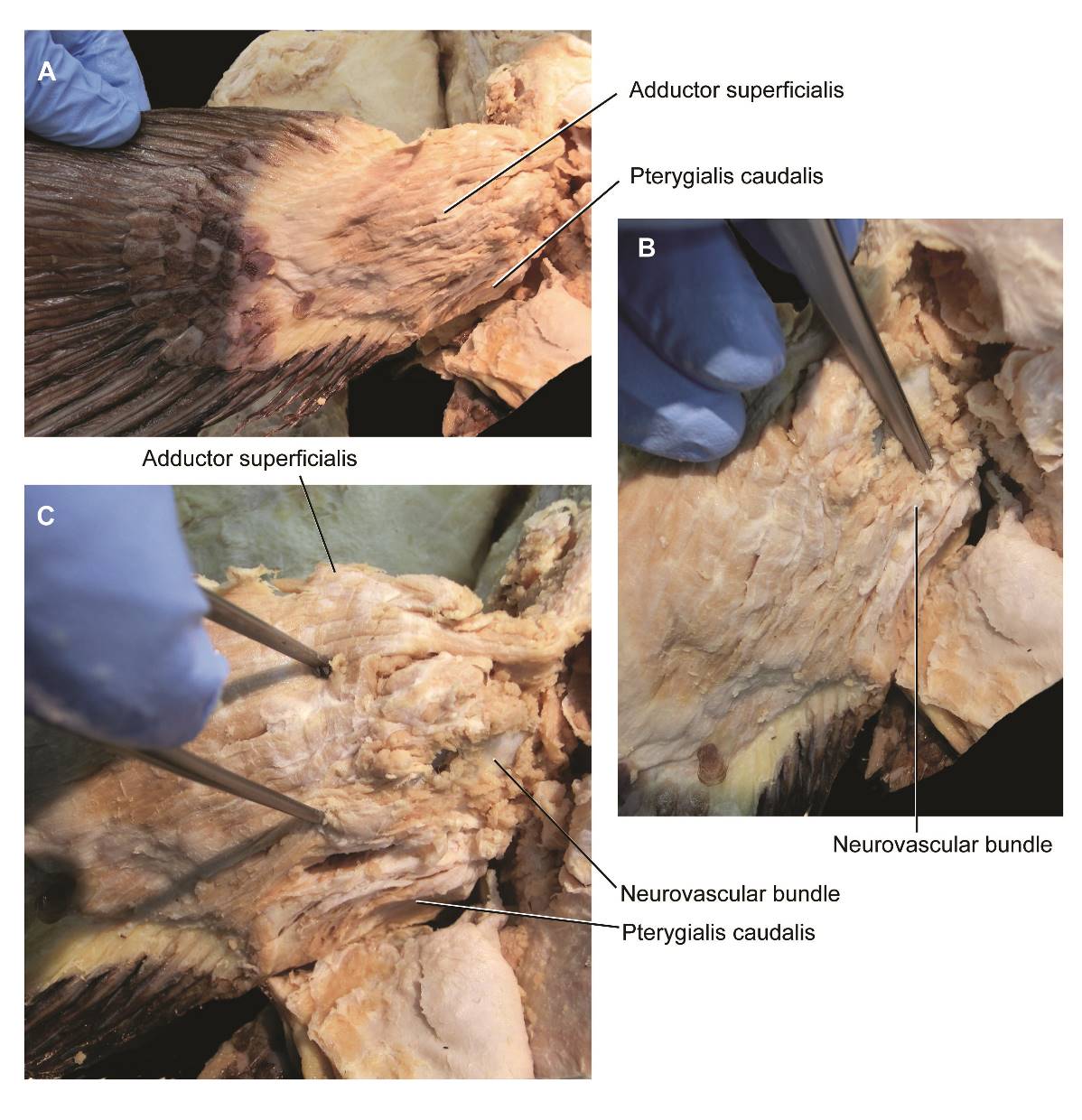
**Fig. S3.** A further example of the type of detailed dissections done for the present work (specific details about the musculoskeletal structures of each appendage of *Latimeria* and *Neoceratodus* are given in the main text and its Tab S1-S6 and Figs. 1-3): a dorsal view of the left pelvic fin of *Latimeria.* A) Intact fin with skin removed; B) close-up of neurovascular bundle separating adductor superficialis and pterygialis caudalis; C) dorsal-caudal view showing close-up of pterygialis caudalis.


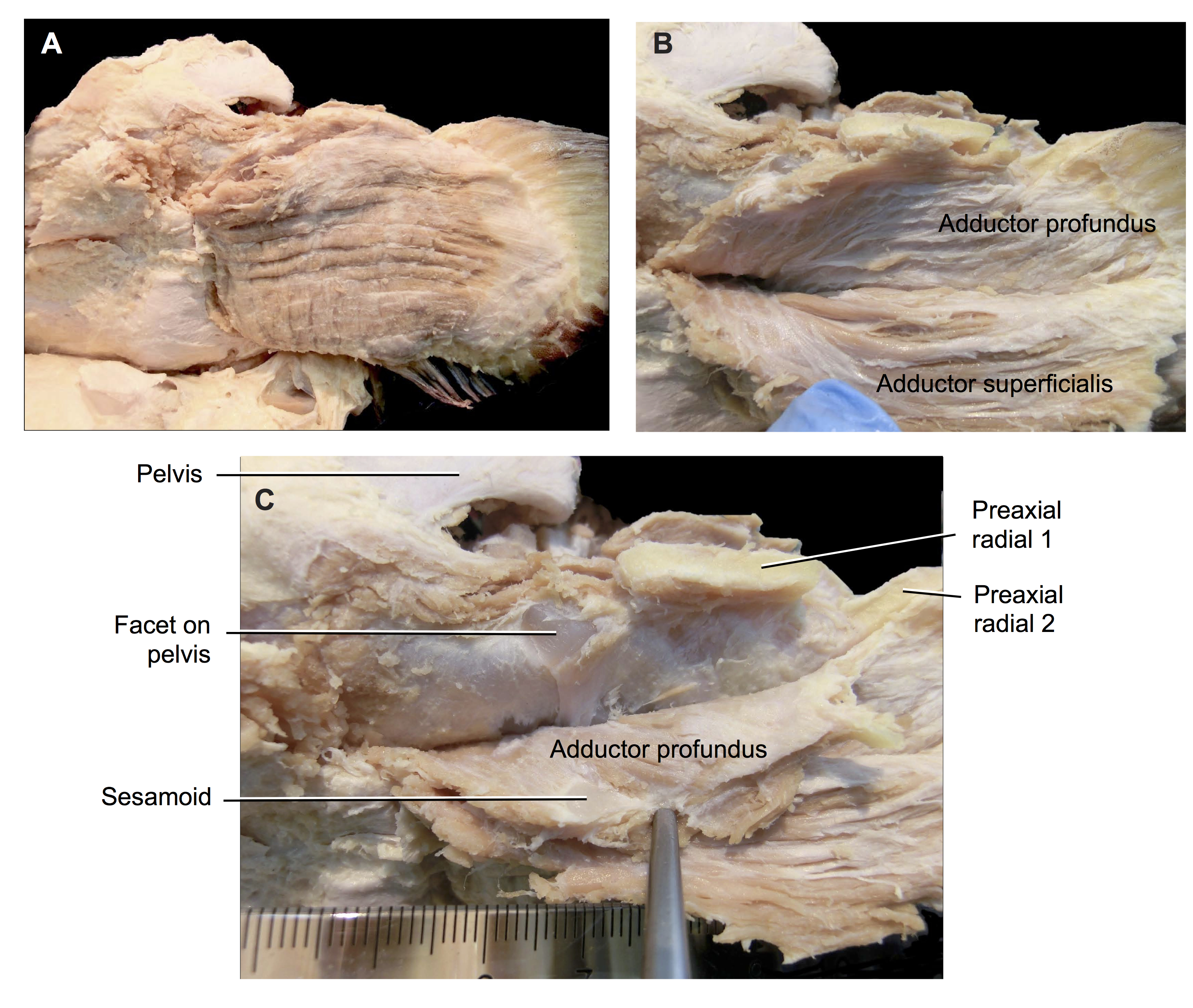
Fig. S4. A last example of the type of detailed dissections done for the present work (specific details about the musculoskeletal structures of each appendage of *Latimeria* and *Neoceratodus* are given in the main text and its Tabs S1-S6 and Figs. 1-3): a ventral view of the left pelvic fin of *Latimeria.* A) Intact fin with skin removed; B) adductor superficialis retracted to show adductor profundus 1; C) adductor profundus retracted to show most proximal preaxial sesamoid bone and its articular facet on the pelvis; D) dorsal view of left pelvic fin showing more distal sesamoid bone and its articular facet on the humerus.


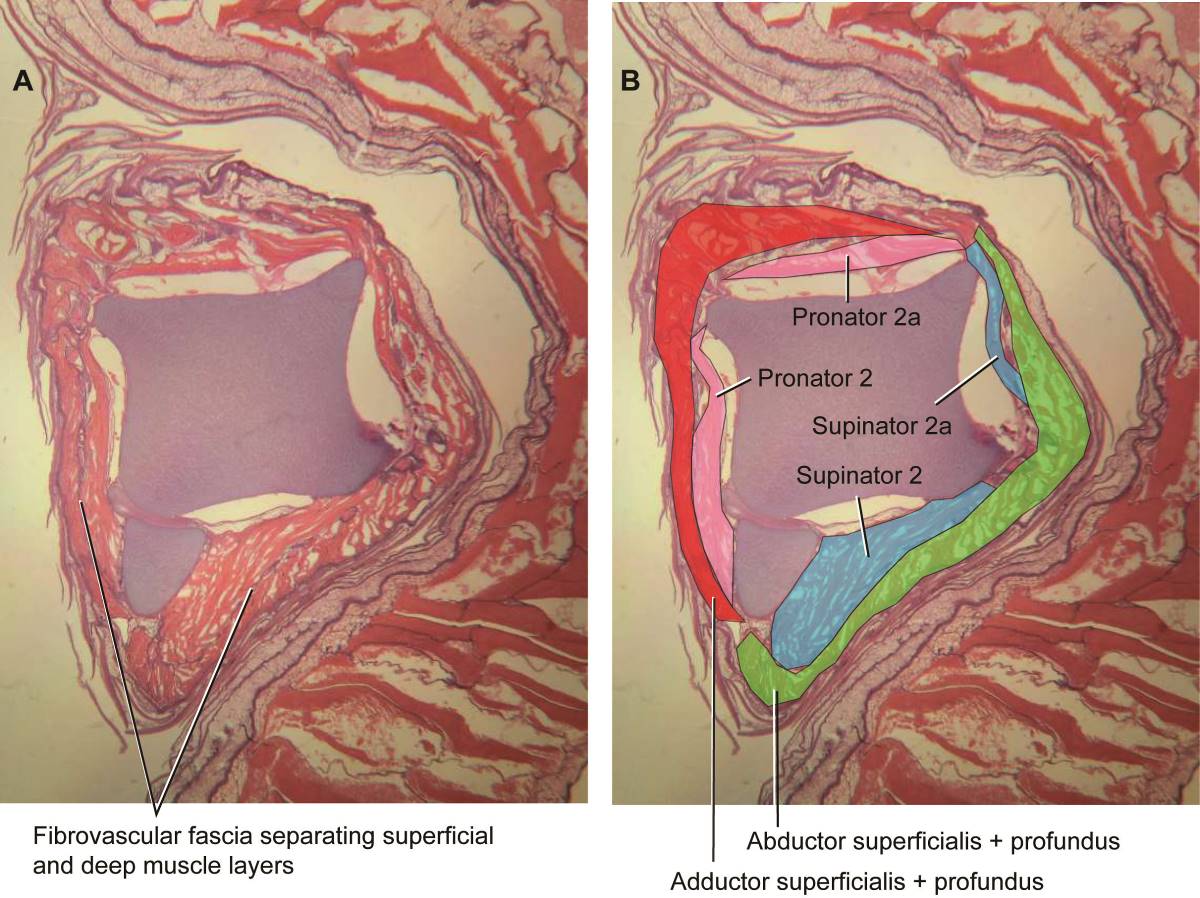


**Fig. S5.** An example of the type of detailed histological sections used in the present work (specific details about the musculoskeletal structures of each appendage of *Latimeria* and *Neoceratodus* are given in the main text and its Tabs S1-S6 and Figs. 1-3): section of the pectoral fin of *Latimeria.* A) Cross-section of humerus showing fibrovascular fascia separating superficial and deep muscle layers; B) same as A but with individual muscles color-coded.


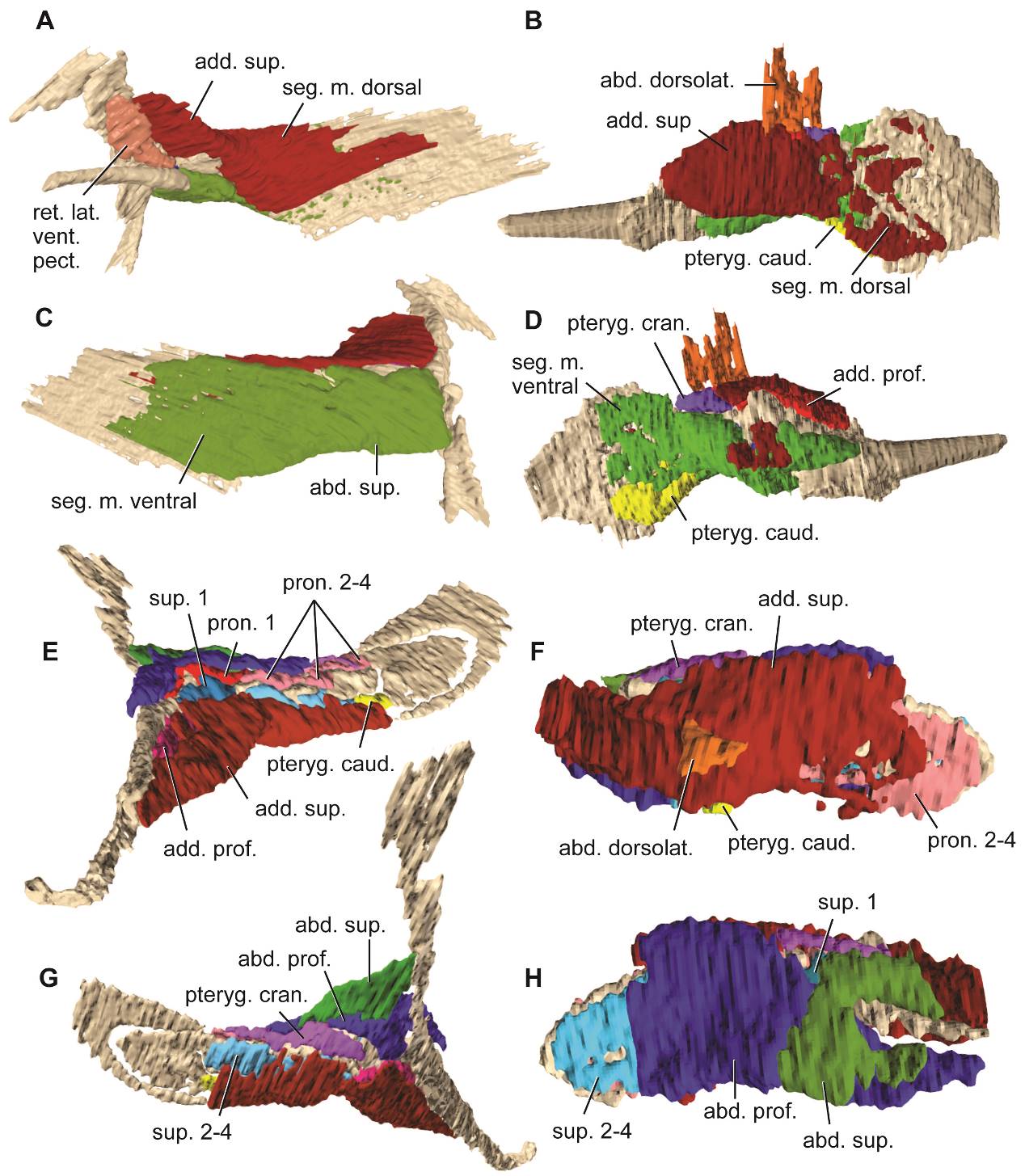


Fig. S6. An example of the type of detailed 3D reconstructions from MRI scans used in the present work (specific details about the musculoskeletal structures of each appendage of *Latimeria* and *Neoceratodus* are given in the main text and its Tab S1-S6 and Figs. 1-3): appendages of *Latimeria* and *Neoceratodus.* A) *Neoceratodus* pectoral fin, dorsal view; B) *Neoceratodus* pelvic fin, dorsal view; C) *Neoceratodus* pectoral fin, ventral view; D) *Neoceratodus* pelvic fin, ventral view; E) *Latimeria* pectoral fin, dorsal view; F) *Latimeria* pelvic fin, dorsal view; G) *Latimeria* pectoral fin, ventral view; H) *Latimeria* pelvic fin, ventral view. Abbreviations: abductor profundus (abd. prof.), abductor superficialis (abd. sup.), abductor dorsolateralis (abd. dorsolat.), adductor profundus (add. prof.), adductor superficialis (add. sup.), pronator (pron.), pterygialis caudalis (pteryg. caud.), pterygialis cranialis (pteryg. cran.), retractor lateralis ventralis pectoralis (ret. lat. vent. pect.) segmented muscle (seg. m.), supinator (sup.). Colors correspond to text figures 1-3.

Table S1. Origins and insertions of pectoral muscles of *Neoceratodus.*

| **Muscle** | **Origin** | **Insertion** |
| --- | --- | --- |
| Retractor lateralis ventralis pectoralis | Cranial rib | Medial face of cleithrum |
| Adductor superficialis (including dorsal superficial segmented muscular layer) | Cleithrum and scapulocoracoid dorsal to articular process | Via aponeurosis onto distal radials and bases of lepidotrichia; divided by tendinous sheets that insert on joints between axial elements |
| Adductor profundus | Scapulocoracoid dorsal to articular process | Dorsal face of 1st element |
| Abductor superficialis (including ventral superficial segmented muscular layer) | Lateral face of clavicle, cleithrum, scapulocoracoid ventral to articular process | Distal radials and bases of lepidotrichia; divided by tendinous sheets that insert on joints between axial elements |
| Abductor profundus | Scapulocoracoid adjacent to and ventral to articular process | Postaxial border of 1st element |

**Table S2.** Origins and insertions of pelvic muscles of *Neoceratodus.* Names in parentheses from*^56^*.

| **Muscle** | **Origin** | **Insertion** |
| --- | --- | --- |
| Abductor dorsolateralis ('superficial ventrolateral abductor') | Body wall muscles dorsal to pelvis | Distal, lateral edge of 1st element |
| Adductor superficialis ('mesial abductor' + superficial dorsal segmented layer that corresponds to 'dorsal lepidrotrichial flexors + radial flexors') | Midline raphe connecting with adductor superficialis on the contralateral side | Distal radials and bases of lepidotrichia; divided by tendinous sheets that insert on joints between axial elements |
| Pterygialis caudalis (postaxial muscle, or 'superficial ventrolateral + ventromesial adductor'') | Midline raphe connecting with pterygialis caudalis on the contralateral side | Distal, medial edge of 1st element |
| Adductor profundus ('dorsomesial adductor-levator') | Dorsal face of pubic ramus | Joint between 1st and 2nd elements via tendinous sheet |
| Pronator 1 (dorsolateral abductor-levator) | Caudolateral face of pubic ramus | Proximal, lateral edge of 1st element and joint between 1st and 2nd elements with adductor profundus |
| Pronators 2-9 (dorsal 'radial-axial' muscles) | All axial elements | Distal ends of radials |
| Abductor superficialis ('superficial ventromesial abductor' + superficial ventral segmented layer that corresponds to 'ventral lepidrotrichial flexors + radial flexors') | Anterolateral process of pelvis and adjacent (lateral) body wall | Distal radials and bases of lepidotrichia; divided by tendinous sheets that insert on joints between axial elements |
| Pterygialis cranialis (preaxial muscle, or part of 'superficial ventromesial abductor') | Caudolateral face of pubic ramus | Distal end of 1st preaxial radial |
| Abductor profundus ('deep ventral abductor-depressor') | Ventrolateral face of pelvis caudal to anterolateral process | Distal, ventral edge of 1st element |
| Supinator 1 ('deep ventral adductor-depressor') | Medial face of pubic ramus | Distal, medial edge of 1st element |
| Supinators 2-9 (ventral 'radial-axial' muscles) | All axial elements | Distal ends of radials |

**Table S3.** Origins and insertions of pectoral muscles of *Latimeria*. Names in parentheses from*^13^*.

| **Muscle** | **Origin** | **Insertion** |
| --- | --- | --- |
| Adductor superficialis ('levator superficialis') | posteromedial border of cleithrum between anocleithrum and endoskeleton | via a broad tendon that onto bases of lepidotrichia; bundles insert onto preaxial radials with pronators 2-3 |
| Adductor profundus ('levator profundus') | medial face of cleithrum and endoskeleton in region of articular process | deep face of adductor superficialis |
| Pronator 1 | medial face of endoskeleton adjacent to articular process | 1st preaxial radial and adjacent joint between 1st and 2nd elements; a bundle continues with pronator 2 |
| Pronator 2 | post axial border of 1st element | 2nd prexial radial and adjacent joint between 2nd and 3rd elements; a bundle continues with Pronators 3 and 4 |
| Pronator 2a | preaxial border of 1st element | with pronator 2 |
| Pronator 3 | post axial border of 2nd element | bases of first 8-10 prexial lepidotrichia |
| Pronator 3a | preaxial border of 2nd element | with pronator 3 |
| Pronator 4 + 4a | pre- and post axial borders of 3rd element | bases of preaxial lepidotrichia distal to pronator 3 and small cartilages distal to 4th element |
| Abductor superficialis ('abaisseur' superficialis) | medial face of cleithrum, extracleithrum and clavicle ventral to the articular process | via a broad aponeurosis onto bases of lepidotrichia; bundles insert onto preaxial radials with supinators 2-3 |
| Abductor profundus ('abaisseur' profundus) | medial face of endoskeleton ventral to articular process | deep face of abductor superficialis |
| Supinator 1 | medial face of endoskeleton immediately adjacent and ventral to articular process | 1st preaxial radial and dorsolateral aspect of the joint between 1st and 2nd elements |
| Supinator 2 | post axial border of 1st element | 2nd prexial radial and adjacent joint between 2nd and 3rd elements; partially fused with supinator 3 |
| Supinator 2a | preaxial border of 1st element | with supinator 2 |
| Supinator 3 | post axial border of 2nd element | bases of first 8-10 prexial lepidotrichia; partially fused with supinators 2 and 4 |
| Supinator 3a | preaxial border of 2nd element | with supinator 3 |
| Supinator 4 | post axial border of 3rd element | bases of preaxial lepidotrichia distal to supinator 3 and small cartilages distal to 4th element; partially fused with supinator 3 |
| Supinator 4a | preaxial border of 3rd element | with supinator 4 |
| Pterygialis caudalis (postaxial muscle, or 'supinator 5 and/or pronator 5') | postaxial borders of 1st - 3rd elements together with pronators and supinators 2-4 | postaxial border between aponeuroses of adductor and abductor superficialis |
| Pterygialis cranialis (preaxial muscle) | from abductor superficialis | preaxial radials and bases of lepidotrichia |

**Table S4.** Origins and insertions of pelvic muscles of *Latimeria*. Names in parentheses from*^13^*.

| **Muscle** | **Origin** | **Insertion** |
| --- | --- | --- |
| Levator lateralis | Fascia of body wall muscles | Preaxial edge of 1st element |
| Adductor superficialis ('levator superficialis') | Dorsal face of lateral process of pelvis | Bases of lepidotrichia via aponeurosis, distal to pronator insertion |
| Pterygialis caudalis (postaxial muscle, or 'pelvic adductor') | Distal extremity of longitudinal shaft of pelvis, passes along postaxial border | Bases of postaxial lepidotrichia |
| Adductor profundus ('levator profundus) | Proximal 2/3 of dorsolateral face of pelvis | Bases of lepidotrichia via aponeurosis, distal to supinator insertion |
| Pronator 1 | Dorsal face of pelvis anterior to articular process | Preaxial cartilages and bases of lepidotrichia; partially fused with pronator 2 |
| Pronator 2 | Postaxial border proximal to lepidotrichia | Preaxial cartilages and bases of lepidotrichia distal to pronator 1; partially fused with pronators 1 and 3 |
| Pronator 3 | Postaxial border proximal to lepidotrichia and distal to pronator 2 | Preaxial cartilages and bases of lepidotrichia distal to pronator 2; partially fused with pronators 2 and 4 |
| Pronator 4 | Postaxial border proximal to lepidotrichia and distal to pronator 3 | Preaxial cartilages and bases of lepidotrichia distal to pronator 3; partially fused with pronator 3 |
| Abductor superficialis ('abaisseur' superficialis) | Ventral face of the pelvis in 2 bundles | Covers the ventral face of the fin, gives way to a broad tendon at the level of the 3-4 element joint, inserts onto bases of lepidotrichia |
| Pterygialis cranialis (preaxial muscle, or 'pelvic abductor') | Ventral face of lateral process of pelvis, passes along preaxial border | Bases of preaxial lepidotrichia |
| Abductor profundus ('abaisseur' profundus) | Medial side of the longitudinal shaft (medial component) of pelvis | Lies deep to abductor superficialis, breaks into poorly defined tendons at a more proximal level than that muscle and these insert into the aponeurosis of that muscle |
| Supinator 1 | Medial border of pelvis at the level of the articular process | Preaxial radials and bases of lepidotrichia; partially fused with supinator 2 |
| Supinator 2 | Postaxial border proximal to lepidotrichia | Preaxial radials and bases of lepidotrichia; partially fused with supinators 1 and 3 |
| Supinator 3 | Postaxial border proximal to lepidotrichia and distal to supinator 2 | Preaxial radials and bases of lepidotrichia distal to supinator 2; partially fused with supinators 2 and 4 |
| Supinator 4 | Postaxial border proximal to lepidotrichia and distal to supinator 3 | Preaxial radials and bases of lepidotrichia distal to supinator 3; partially fused with supinator 3 |

**Table S5.** Our interpretations of the homologies between the muscles (including synonyms) of the pectoral appendage of the shark *Squalus* (Chondrichthyes), the bichir *Polypterus* (Actinopterygii: Cladistia) and of Sarcopterygii: the coelacanth *Latimeria* (Coelacanthimorpha), lungfish *Neoceratodus* (Dipnomorpha) and salamander *Ambystoma* (Tetrapoda).

| **Muscle groups** | ***Squalus* (5 muscles)** | ***Polypterus* (6 muscles)** | ***Latimeria* (20 muscles)** | ***Neoceratodus* (5 muscles)** | ***Ambystoma* (28 muscles; 48 with hand muscles)** | |
| --- | --- | --- | --- | --- | --- | --- |
| Primaxial musculature | - (retractor lateralis ventralis pectoralis poorly differentiated) | - (retractor lateralis ventralis pectoralis poorly differentiated) | - (retractor lateralis ventralis pectoralis seemingly undifferentiated) | Retractor lateralis ventralis pectoralis ('muscle connecting cranial rib to girdle') | Serratus anterior | |
|  |  |  |  |  | Levator scapulae | |
| Adductor superficialis (superficial dorsomesial musculature) | Adductor superficialis | Adductor superficialis | Adductor superficialis ('levator superficialis') | Adductor superficialis (including dorsal superficial segmented muscular layer) | Deltoideus scapularis | |
|  |  |  |  |  | Latissimus dorsi (or it is instead homologous with 'levators 2/3' and/or 'retractor dorsalis pectoralis' of e.g., some chondrichthyans (as it is single appendicular muscle with both abaxial & primaxial developmental features)? | |
|  |  |  |  |  | Part of triceps (i.e., triceps scapularis & triceps humeralis lateralis; and perhaps triceps medialis?) | |
|  |  |  |  |  | Extensor digitorum | |
|  |  |  |  |  | Extensor carpi radialis + supinator | |
|  |  | Pterygialis caudalis (postaxial muscle, or 'dilatator posterior' or 'coracometapterygialis I-II) | Pterygialis caudalis (postaxial muscle, or 'supinator 5 and/or pronator 5') |  | Extensor antebrachii et carpi ulnaris | |
|  |  |  |  |  | Part of triceps (i.e., triceps coracoideus) | |
| Adductor profundus (deep dorsomesial musculature) | Adductor profundus | Adductor profundus | Adductor profundus ('levator profundus) | Adductor profundus | Procoracohumeralis | |
|  |  |  | Pronator 1 |  | Subcorascapularis | |
|  |  |  | Pronator 2 |  | Abductor et extensor digit 1 | |
|  |  |  | Pronator 2a |  | Extensores breves digitorum 2-4 | |
|  |  |  | Pronator 3 |  |  |  |
|  |  |  | Pronator 3a |  |  |  |
|  |  |  | Pronator 4 + 4a |  |  |  |
| Abductor superficialis (superficial ventrolateral musculature) | Abductor superficialis | Abductor superficialis | Abductor superficialis ('abaisseur' superficialis) | Abductor superficialis (including ventral superficial segmented muscular layer) | Pectoralis | + some/all intrinsic hand muscles? |
|  |  |  |  |  | Flexor digitorum communis |  |
|  |  |  |  |  | Flexor antebrachii et carpi ulnaris |  |
|  |  |  |  |  | Coracobrachialis |  |
|  | Pterygialis cranialis (preaxial muscle) | Pterygialis cranialis (preaxial muscle, or 'dilatator anterior' or 'zonopropterygialis') | Pterygialis cranialis (preaxial muscle) |  | Flexor antebrachii et carpi radialis |  |
|  |  |  |  |  | Humeroantebrachialis |  |
| Abductor profundus (deep ventrolateral musculature) | Abductor profundus | Abductor profundus | Abductor profundus ('abaisseur' profundus) | Abductor profundus | Supracoracoideus | |
|  |  |  | Supinator 1 |  | Coracoradialis | |
|  |  |  | Supinator 2 |  | Flexor accessorius medialis | + some/all intrinsic hand muscles? |
|  |  |  |  |  | Palmaris profundus 1 |  |
|  |  |  |  |  | Pronator quadratus |  |
|  |  |  | Supinator 2a |  | - |  |
|  |  |  | Supinator 3 |  | Flexor accessorius lateralis |  |
|  |  |  | Supinator 3a |  | - |  |
|  |  |  | Supinator 4 |  | Contrahentium caput longum |  |
|  |  |  | Supinator 4a |  | - |  |

**Table S6.** Our interpretations of the homologies between (including synonyms of) the muscles of the pelvic appendage of the shark *Squalus* (Chondrichthyes), the bichir *Polypterus* (Actinopterygii: Cladistia) and of Sarcopterygii: the coelacanth *Latimeria* (Coelacanthimorpha), the lungfish *Neoceratodus* (Dipnomorpha) and the salamander *Ambystoma* (Tetrapoda).

| **Muscle groups** | ***Squalus* (5 muscles)** | ***Polypterus* (6 muscles)** | ***Latimeria* (15 muscles)** | ***Neoceratodus* (25 muscles)** | ***Ambystoma* (27 muscles; 59 with foot muscles)** | |
| --- | --- | --- | --- | --- | --- | --- |
| Abaxial, and partially primaxial? (because these muscles originate proximally from axial skeleton and/or musculature) | - | - | Levator lateralis | Abductor dorsolateralis ('superficial ventrolateral abductor') | Caudofemoralis (included here because of origin from axial skeleton/muscles, but direct homology with *Latimeria*'s lateral levator and/or *Neoceratodus*' dorsolateral abductor is not assumed, as e.g., the muscle of *Latimeria* is seemingly part of dorsal musculature, while caudofemoralis is part of ventral musculature) | |
| Adductor superficialis (superficial dorsomesial musculature) | Adductor superficialis | Adductor superficialis | Adductor superficialis ('levator superficialis') | Adductor superficialis ('mesial adductor' + superficial dorsal segmented layer that corresponds to 'dorsal lepidrotrichial flexors + radial flexors') | Extensor iliotibialis ('iliotibialis') | |
|  |  |  |  |  | Extensor cruris tibialis | |
|  |  |  |  |  | Extensor tarsi tibialis | |
|  |  |  |  |  | Extensor digitorum longus | |
|  |  | Pterygialis caudalis (postaxial muscle: present in our microCT scans and dissections of *Polypterus*) | Pterygialis caudalis (postaxial muscle, or 'pelvic adductor') | Pterygialis caudalis (postaxial muscle, or 'superficial ventrolateral + ventromesial adductor'') | Extensor cruris et tarsi fibularis | |
|  |  |  |  |  | Tenuissimus ('iliofibularis') | |
| Adductor profundus (deep dorsomesial musculature) | Adductor profundus | Adductor profundus | Adductor profundus ('levator profundus) | Adductor profundus ('dorsomesial adductor-levator') | Puboischiofemoralis internus | |
|  |  |  | Pronator 1 | Pronator 1 | Iliofemoralis | |
|  |  |  | Pronator 2 | Pronators 2-9 (dorsal 'radial-axial' muscles) | Abductor et extensor digit 1 | |
|  |  |  | Pronator 3 |  | Extensores breves digitorum 2-5 | |
|  |  |  | Pronator 4 |  |  |  |
| Abductor superficialis (superficial ventrolateral musculature) | Abductor superficialis | Abductor superficialis | Abductor superficialis ('abaisseur' superficialis) | Abductor superficialis ('superficial ventromesial abductor' + superficial ventral segmented layer that corresponds to 'ventral lepidrotrichial flexors + radial flexors') | Gracilis ('puboischiotibialis') | + some/all intrinsic foot muscles? |
|  |  |  |  |  | Flexor digitorum communis |  |
|  |  |  |  |  | + Pubotibialis? (or pubotibialis derived from pterygialis cranialis or, more likely, from abductor profundus?) |  |
|  | Pterygialis cranialis (preaxial muscle, or 'pelvic protractor') | Pterygialis cranialis (preaxial muscle, or 'dilatator anterior') | Pterygialis cranialis (preaxial muscle, or 'pelvic abductor') | Pterygialis cranialis (preaxial muscle, or part of 'superficial ventromesial abductor') | Ischioflexorius (which likely includes flexor cruris et tarsi tibialis) and perhaps femorofibularis (+ pubotibialis? see above) |  |
| Abductor profundus (deep ventrolateral musculature) | Abductor profundus | Abductor profundus | Abductor profundus ('abaisseur' profundus) | Abductor profundus ('deep ventral abductor-depressor') | Puboischiofemoralis externus + adductor femoris (pubofemoralis') (+ pubotibialis? see above) | |
|  |  |  | Supinator 1 | Supinator 1 ('deep ventral adductor-depressor') | Ischiotrochantericus ('ischiofemoralis') | |
|  |  |  | Supinator 2 | Supinators 2-9 (ventral 'radial-axial' muscles) | Flexor accessorius medialis | + some/all intrinsic foot muscles? |
|  |  |  |  |  | Tibialis posterior ('pronator profundus') |  |
|  |  |  |  |  | Interosseus cruris |  |
|  |  |  | Supinator 3 |  | Flexor accessorius lateralis |  |
|  |  |  | Supinator 4 |  |  |  |
|  |  |  |  |  | Contrahentium caput longum |  |

**Table S7.** Our interpretations of topological correspondences between forelimb and hindlimb muscles in salamanders and in humans, based on the information provided in Tables S5 and S6. Names of muscles shown in bold highlight cases in which there is a one-to-one muscle correspondence in fore-hindlimb of salamanders (blue) and in fore-hindlimb of humans (red).

| **Muscle groups** | ***Ambystoma* forelimb** | ***Ambystoma* hindlimb** | ***Homo* forelimb** | ***Homo* hindlimb** | **Notes on human fore-hindlimb comparison** |
| --- | --- | --- | --- | --- | --- |
| Primaxial | - | - (or quadratus lumborum present?) | - | Quadratus lumborum | - |
|  | Serratus anterior + Levator Scapulae | - | Serr. ant.+Lev. scapulae + Rhomboideus major & minor + Subclavius | - | triceps=quadriceps & deltoideus = gluteus maximus supported by Coues 1872; latissimus dorsi = gluteus maximus by Humphry 1872 and Quain 1894; deltoideus = sartorius by Humphry 1872 |
| Abaxial+partially primaxial? | - | Caudofemoralis | - | - |  |
|  | - (Latissimus dorsi?) | - | - (Latissimus dorsi?) | - |  |
| Adductor superficialis | Deltoideus scapularis + Latissimus dorsi (?) + Part Triceps brachii (e.g., scapularis) | Extensor iliotibialis | Part of Deltoideus + Lat. dor. (?) + Part of Triceps (e.g., long head) | Gluteus maximus + Quadriceps femoris + Sartorius |  |
|  | **Extensor digitorum** | **Extensor digitorum longus** | Extensor digitorum | Ex. dig. & hallucis longus + Fib. tertius | Supported by Diogo et al. 2013 |
|  | **Supinator** | **Extensor cruris tibialis** | Supinator | Part of Tibialis anterior | Supported by Diogo et al. 2013 |
|  | **Extensor carpi radialis** | **Extensor tarsi tibialis** | Ex. ca. ra. br. & lon. + Brachioradialis | Part of Tibialis anterior | Supported by Diogo et al. 2013 |
| Pterygialis caudalis (postaxial) | **Extensor antebrachii et carpi ulnaris** | **Extensor cruris et tarsi fibularis** | Extensor carpi ulnaris + anconeus | Fibularis longus & brevis | Supported by Diogo et al. 2013 |
|  | Part of Triceps brachii (i.e., t. coracoideus) | Tenuissimus ('iliofibularis') | Part of Triceps brachii | Part of Biceps Femoris | Ontogeny: tenuissimus migrates to ventral side to fuse with biceps fem. |
| Adductor profundus | **Procoracohumeralis** | **Puboischiofemoralis internus** | Part of Deltoideus + Teres minor | Iliopsoas + Part of Pectineus | Supported by Humphry 1872 |
| Pronator 1 | **Subcorascapularis** | **Iliofemoralis** | Subscapularis + Teres major | Glu. med. & min. + Pirif.. +Ten. fas. lat. | T.ma.=part of iliofem. by Coues 1872;sub.= glut.med&min.+pir.+t.f.l. by Quain 1894 |
| Other pronators | **Abductor et extensor digit 1** | **Abductor et extensor digit 1** | Abd. pol. longus +Ex. pol. bre. &long. | Ex. hallucis brevis | Supported by Diogo et al. 2013 |
|  | **Extensores breves digitorum 2-4** | **Extensores breves digitorum 2-5** | Ex.indicis + Ex. digiti minimi | Extensor digitorum brevis | Supported by Diogo et al. 2013 |
| Abductor superficialis | Pectoralis + Coracobrachialis | Gracilis ('puboischiotibialis') + Pubotibialis (?) | Pect. major & minor + Coracobr. + Part of Biceps brachii | Gracilis + Ad. long. + Part of Pectineus | pect.=pectin. & coracob.=adductors & part of bi. bra. = graci. by Coues 1872; pect.=graci. +add. lon. + part of pect. by Humphry 1872 and Quain 1894 |
|  | Flexor digitorum communis + Flexor antebrachii et carpi ulnaris | Flexor digitorum communis | **Fl. dig. profundus** + **Fl. pollicis longus** + **palmaris longus** + Fl. carpi ulnaris + part of Fl. dig. superficialis | **Fl. dig. longus** + **Fl. hallucis longus** + **Plantaris** + Soleus + Part of Quadr. plantae + Gastrocnemius | Supported by Diogo et al. 2013 |
| Pterygialis cranialis (preaxial) | Flexor antebrachii et carpi radialis + Humeroantebrachialis | Ischioflexorius (which likely includes flexor cruris et tarsi tibialis) + Femorofibularis (?) | Fl. car. radi. + Pronat. ter. + Brachialis (+ part of Bic. brachii, if partially derives from humeroantebrachialis?) | Semimembranosus + Semitendinosus + Part of Biceps femoris + Part of Adductor magnus | bic. br.=bic. fe. & brachialis = semiten. + semimem. by Coues 1872 & rough-ly by Quain 1894 and Humphry 1872 |
| Abductor profundus | Supracoracoideus | Puboischiofemoralis externus + adductor femoris (pubofemoralis') | Infraspinatus + Supraspinatus | Obtur. externus + Quadratus fem. + Ad. brevis + Part of Ad. magnus | Ontogeny: mostly ventral muscles that seem to move dorsally, supporting correspondence |
| Supinator 1 | **Coracoradialis** | **Ischiotrochant.** ('ischiofemoralis') | Part of Biceps brachii | Obtur. internus + gemellus inf. &sup. | Supported by Diogo et al. 2013 |
| Other supinators | **Flexor accessorius medialis** | **Flexor accessorius medialis** | - (Part of fl. dig. profundus) | - (Part of Fl. dig. longus & of Qua. plant.) | Supported by Diogo et al. 2013 |
|  | **Palmaris profundus 1** | **Tibialis posterior ('prona. profundus')** | - | Tibialis posterior | Supported by Diogo et al. 2013 |
|  | **Pronator quadratus** | **Interosseus cruris** | Pronator quadratus | Popliteus | Supported by Diogo et al. 2013 |
|  | **Flexor accessorius lateralis** | **Flexor accessorius lateralis** | - (Part of fl. dig. profundus) | - (Part of Fl. dig. longus) | Supported by Diogo et al. 2013 |
|  | **Contrahentium caput longum** | **Contrahentium caput longum** | - (Part of fl. dig. profundus) | - (Part of Fl. dig. longus) | Supported by Diogo et al. 2013 |
| Intrinsic autopod muscles in tetrapods | **Flexores breves superficiales** | **Flexores breves superficiales** | Part of **Fl. dig. sup**. + Palmaris brevis | **Flexor digitorum brevis** | Supported by Diogo et al. 2013 |
|  | **Contrahentes digitorum** | **Contrahentes pedis** | **Add. pollicis** + **Ad. pol. accessorius** | **Add. pollicis** + **Ad. hal. accessorius** | Supported by Diogo et al. 2013 |
|  | **-** | **-** | **Lumbricales** | **Lumbricales** | Supported by Diogo et al. 2013 |
|  | **Flexores breves profundi** | **Flexores breves profundi** | **Fl. pol. br.** & **dig,. min**. + Oppo.. pol. & di. mi. + **Int. palm.** + Part of **Int.. dor.** | **Fl. hal. br.** & **dig,. min.** + **Int. plant.** + Part of **Int. dorsales** | Supported by Diogo et al. 2013 |
|  | **Fls. digitorum minimi +Interphalangeus 3** | **Fls. digito. min. +Interphalangei 3-4** | - | - | Supported by Diogo et al. 2013 |
|  | **Abductor digiti minimi** | **Abductor digiti minimi** | **Abductor digiti minimi** | **Abductor digiti minimi** | Supported by Diogo et al. 2013 |
|  | **-** | **-** | **Abductor pollicis brevis** | **Abductor hallucis** | Supported by Diogo et al. 2013 |
|  | **Intermetacarpales** | **Intermetacarpales** | Part if **Interossei dorsales** | Part if **Interossei dorsales** | Supported by Diogo et al. 2013 |
